# Hypodermic Use of Carbolic Acid for the Cure of Erysipelas

**Published:** 1884-07-20

**Authors:** J. McF. Gaston

**Affiliations:** Atlanta, Georgia


### Southern Medical Record:

EDITORS:
THOMAS S. POWELL, M.D. R. C. WORD, M.D.

*R. C. WORD, M.D., Managing Editor.*

☞All Communications and Letters on Business connected with the RECORD must be addressed to the Managing Editor.

VOL. XIV. ATLANTA, GA., JULY 20, 1884. No. 7.

### ORIGINAL AND SELECTED ARTICLES.

### HYPODERMIC USE OF CARBOLIC ACID FOR THE CURE OF ERYSIPELAS.

BY J. McF. GASTON, M.D., ATLANTA, GEORGIA.

As I am not aware of the publication by any other observer in the United States of facts connected with the subcutaneous injection of carbolic acid in erysipelas, it is hoped that a brief detail of my experience with this treatment may attract the attention of practitioners to the great virtues of this remedy.

I was induced to resort to this measure originally by the recommendation of it which appeared September 7th, 1878, in the "Medical Times and Gazette" of London; but not laying so much stress upon great names as upon great works, my note book gives no record of the authorship. The formula given was as follows:

℞ Carbolic acid,<br>

Spirits of wine, } ....................aa 1.5 grammes,<br>

Distilled water.......................... 50 grammes.

Mix and inject in various points of the surface affected with erysipelas.

Having in the meantime employed a different formula in various other affections with a very satisfactory result, I concluded to adopt the following in preference to using the above in cases of erysipelas which might come under my care:

℞ Fluid carbolic acid.........................gtts. xx,

Glycerine...............................ʒ ij,

Distilled water..........................ʒ vi.

The first case treated by this process was upon the upper part of the cheek, and extending to the ear and eyebrow on the same side. A single syringeful of the last named combination was inserted above the prominence of the malar bone on two successive days, without using any other means of treatment, and the erysipelatous blotch disappeared entirely. This case was followed by others in which considerable portions of the surface were involved, upon various parts of the body, with a like satisfactory issue in all. It was evident that the speedy disappearance of the discoloration and thickening of the skin, which accompanies erysipelas, was not simply a coincidence, but that it was due to the favorable action of the subcutaneous injection. With a view to determine more certainly the relation of cause and effect, quite a number of cases of localized and circumscribed patches of erysipelas were treated solely with the hypodermic application of carbolic acid. Among other cases of the ankles and legs treated successfully in this manner, there occurred a remarkably striking illustration of the advantages of this remedy in a chronic case of erysipelas of the lower extremities. For a long series of years this patient had suffered from the frequent recurrence of the disease; and on this occasion both legs were much swollen and the tissues presented the well marked characteristics of erysipelas from the knees to the ankles. With the history of his diathesis, it seemed doubtful as to his being relieved as others had been in the acute form of more recent origin. But he was influenced by the favorable reports from other cases to undertake the slight operation of inserting the carbolic acid solution upon the outer and inner aspect of each leg for three consecutive days. Strange as it may seem, without any other treatment, he has passed five years since this application without even a vestige of erysipelas in his legs or any other part of his body, and it served as a radical cure of an established erysipelas diathesis.

It is well known that erysipelas involving the hairy scalp may be transmitted to the brain, and thus becomes one of the gravest of disorders. The phlegmonous termination of erysipelas, in any part of the body, is a most serious development, which has proved very intractable under the ordinary modes of treatment. A remedy that may be considered as trustworthy in averting all the dangers of such a disease is a great advance in therapeutics, and such is the combination above given. Mix and inject one or two fillings of the hypodermic syringe in several points of the surface.

In a paper on the "Innovations of Medical and Surgical Practice," which appeared August 19th, 1882, in the Philadelphia Medical and Surgical Reporter, I used the following language: "But the pre-eminence given latterly to the use of carbolic acid by subcutaneous injection claims something more than a passing notice. Thus applied, it effectually arrests erysipelas."

An article contributed by me to the March number of Gaillard's Journal of the current year upon "Medication Through Superficial and Deep seated Tissues," contains the following statements: "The hypodermic use of carbolic acid has been resorted to in a variety of cases, and I have been much encouraged to continue this application for local and general effects." * * * "The highly salutary influence of the sub cutaneous use of this agent in erysipelas prompts me to urge the great importance of resorting generally to this mode of cure. The invariable good results that have attended its application in my hands seem to recommend it as a safe and efficacious remedy in erysipelas. In the cases of circumscribed patches the injections have been made in one or two points, according to the extent of the reddening and thickening of the skin; and the local inflammation has in every instance been controlled without recourse to general measures of treatment. In other cases of a generally diffused discoloration I have used the carbolic acid injection accompanied with internal remedies, so that an element of doubt enters as to what brought about the salutary result. One of the cases of erysipelas that involved important parts and a considerable extent of surface was associated with puerperal fever. It was evident that the hypodermic use of the carbolic acid was salutary, and I consider it a most fortunate issue, in consideration of the circumstances, that no phlegmonous inflammation was developed."

It may be questioned as to the applicability of this means to those cases of erysipelas which involve large portions of the surface, and which may be regarded as constitutional in their origin or, at least, general in their character. But, if it is recollected that the introduction of the medicinal agent into the areolar tissue ensures its propagation throughout the organization, it will at once be evident that the measure is not local in its effects, but extends to the entire system. There is an intimate relation between the minute ramifications of the nerves and the capillaries, through which hypodermic injections reach the vital functions and operate upon the various organs and their special secretions, so that there is a general pervading constitutional modification, corresponding to the quality of the medicinal element employed.

The routine treatment of erysipelas is based upon the supposition of a dyscrasia of the blood, and the most trustworthy internal remedy is, perhaps, the muriated tincture of iron in large doses, frequently repeated. It has also been claimed that the progress of the disorder is arrested by encircling the discoloration with a tracing of lunar caustic. Sundry other internal and external means have been adopted in accordance with the views of different pathologists as to the nature of the disease.

Let the influence of subcutaneous injections of carbolic acid in the case of erysipelas be explained as it may, the fact cannot be ignored that many have been promptly relieved by this simple application without the use of any other remedy.

